# Making economic citizens beyond neoliberalism: Historical trajectories of a banker association’s efforts in economic education

**DOI:** 10.1177/14749041251388192

**Published:** 2025-11-11

**Authors:** Thomas Ruoss

**Affiliations:** Swiss Federal University for Vocational Education and Training, Switzerland

**Keywords:** Economic education, citizenship education, international organisation, history of education, privat interest groups

## Abstract

This paper explores the rationales of citizenship that underlie the development of economic education. To achieve this, it analyses the activities of a globally active private interest group, the International Thrift Institute (ITI), focusing on three selected historical periods characterized by crises and change. The analysis shows that the education of economic citizens is a history of juxtapositions of underlying conflicts wrapped in a constant need for consensus to stabilize changing political systems. During crises, the ITI championed different, and conflicting, rationales of economic citizenship. These range from the idea of economic education as an experience-based activity that strengthens individuals’ emotional reflexes in dealing with money, thereby promoting social cohesion, to the idea of economic education as a transformative intervention that creates economic citizens as customers of institutionalized saving services. The paper argues that economic education, given its normative goals, should not be reduced by private interest groups to legitimise and stabilise their agenda. Education for economic citizenship must remain controversial to raise questions about the economy. These are issues that cannot be addressed through individual consumer behaviour, but rather through political action. Economic citizens need to recognise political agency, collective deliberation and structural conditions.

## Education, citizenship and economics in historical perspective: an introduction

In the development of modern societies, education, citizenship and economics have been closely linked, shaping a system in which citizens’ economic knowledge, financial power and economic performance are closely tied to their status as citizens (Ruyskensvelde and Ketch, 2018; Schreiber, 2014). The 20th century, with its political and economic upheavals and transformations, saw repeated renegotiations of the relationship between education, democracy and economics. The idea of the consumer was the one that gain most traction, becoming central to a concept of citizenship that is adaptable to different political contexts and pedagogical concepts (Arthur, 2012; Labaree, 2011; Ricci et al., 2016; Spring, 2003; Streeck, 2012). The relationship of the economic citizen as consumer to society, unlike that of the political subject, is not defined in terms of democratic rights and social integration, but primarily by the possibility of economic interaction and the accumulation of (human) capital. The establishment of this rationale of the economic citizen as an individual consumer, however, was historically contingent – it still is. It is by no means without alternatives. On the contrary, it has always been contested.

The question of the fundamental normativity of citizenship extends beyond the economic content of education (Giudici et al., 2025; Myers & Rapoport, 2021). The tension between conflict and consensus surrounding citizenship education becomes particularly acute in times of political or economic crisis. Such crises are typically accompanied by a decline in trust in political institutions, which, in turn, results in more nationalistic approaches to citizenship, as well as a shift in understandings of citizenship that focus more on individual skills and employability (Hoskins et al., 2016). Recent work also identifies, and criticizes, such crisis-induced effects in relation to the education of economic citizens. Meylemans et al. (2023) attribute such an influence on the 2008 financial crisis, hailing it as an ‘economic shift in thinking about youth citizenship’ (968). More specially, Willis (2017) argues that the financial crisis situation created a strong and normative demand for ‘financial literacy education’. Consequently, international organisations such as the European Commission and the OECD began promoting initiatives to educate those who had recently lost their economic basis, encouraging them to personally manage their money more rationally.

Scholars’ criticism of the idea that economic citizenship should revolve around personal responsibility for managing money needs to be put into perspective. Indeed, both the criticism and their target are not new. The 2008 crisis was just one of several that Western societies have experienced, meaning we need to look further back in time to understand the dynamics that have shaped and re-shaped understandings of economic citizenship. To identify the 2008 crisis as the sole determinant of the current dominant understanding of economic citizenship and to link this understanding to neoliberal ideology, would obscure our understanding of the current controversies surrounding economic citizenship. Against this backdrop, a historically informed perspective can highlight the diversity, contradictions, stability and variability of such citizenship concepts, while also revealing the roles played by both official policymakers and actors operating outside institutional channels of power in these deliberations.

What constitutes an economic citizen is shaped by historical developments and regional traditions, which help to explain contemporary policy disputes around the role and content of economic education. Consequently, diverse rationales of economic education continue to influence how young people learn about markets, politics, and citizenship (Ruoss et al., 2023). A closer look at curricular approaches to economic education developed by social scientists reveals different interpretations that have emerged over time and remain politically contentious to this day. This variety is a consequence, first, of the development of economic ideas. The 20th century alone, for example, saw the rise of neoclassicism, Keynesianism, and neoliberalism, each of which imagined a different role for citizens in the economy (Cohen and Emmett, 2012). Neoliberalism as the ideological underpinning for an individualistic concept of financial literacy is already embedded in a long tradition of ideas and practices in economic education. Second, different disciplines have engaged with economic education, advancing different curricular principles to model the competencies that economic education should encompass (Ackermann, 2021). However, beyond the debate between different approaches to competence modelling, some scholars argue that the tendency to transform economic citizenship into competencies is problematic. From their perspective, we are facing an emerging technocratic model of citizenship education that transforms a normative idea of citizenship into a narrow skills agenda (Joris et al., 2022). They instead call for a broader conception of citizenship and economic citizenship that recognises political agency, collective deliberation and structural conditions—elements that are largely absent from the competence discourse.

Meylemans et al. (2023) call for an analysis of citizenship education concepts in Europe that goes beyond a national perspective. This paper follows their call by focusing not on actors operating at a national or supra-national level but on a private international actor. It shows that citizenship concepts with a specific economic focus emerged long before a supra-national European level actor became involved in debates on citizenship education. At the same time, the debate surrounding citizenship education is not primarily conducted through state regulation. Instead, it is conducted through ‘soft power’ and thus the influence of actors using informational and organisational tools focused on dialog and coordination of activities from the bottom up (Abs, 2021). Therefore, this paper focuses on the importance of actors in the normative debate on what should be taught about, by and through economics. The morally charged field of economics education has been promoted more than any other by the involvement of private lobby groups and associations, rather than by state actors (Ackermann, 2018; Chatel, 2015; Hedtke, 2018; Martinache, 2018; Sukarieh and Tannock, 2009).

Drawing on this work, this paper assumes not only that both the role of schooling and the concept of citizenship have always been politically contested, but also that interest groups played an important role in debates around economic citizenship. Focusing on the educational policies of specific interest groups can help to trace the lines of conflict around education, citizenship and economics. More specifically, this article sheds new light on the history of economic citizenship by engaging, for the first time, with a private international actor founded with a specific mission and commitment to economic education: the International Thrift Institute (ITI). Founded in 1924, the ITI represents a global umbrella organisation of national savings banks that have based their raison d’être on their role in economic education. Because savings banks across Europe have used the ITI as a reference, especially in times of crisis, a historical analysis of this international actor is particularly valuable. This paper therefore explores the following key question: Which rationales of economic citizenship were put forward by the ITI as a global banker association in times of change and crisis from 1920s–1970s?

A historical analysis does not simply focus on the development of a clearly defined contemporary concept; it also considers the emergence of rationales for economic citizenship within specific historical contexts. To identify different rationales of economic citizenship advanced in such contexts, the paper adopts a broad concept of citizenship education, which varies depending on the time, the actor’s perspective and the target audience. Joris (2022) distinguishes between three rationales of citizenship education that each seeks different aims. A first rationale surrounding socialization, which aims to promote social cohesion; a second rationale focussing subjectification, which strives for individual-emancipatory aims; and a rational of individual qualification, which contributes to the further development of the economy and society for transformational aims.

This triadic conceptualisation is particularly useful for this research, because it enables rationales to different social domains and their related aim. The political domain is linked to socialisation aims, the pedagogical domain to individual emancipation aims, and the economic domain to transformational aims. This broad understanding of the pedagogical activities of a private interest group allows for a differentiated analysis. Such a broad understanding of citizenship education and of ‘holistic economic citizenship’ (Crowley & Swan, 2018: 13) has also been put forward to contrast the seemingly narrow understanding of economic citizenship as personal financial responsibility. ‘Financial citizenship’, in Khalil’s (2021, p. 9) suggestion, should be deliberately expanded to include aspects of participation and social justice, in the sense of a ‘holistic, ethical and evolutionary response (. . .) that enables young people to tackle financial, social, and economic systems so they recognize that their economic and financial reality is a political construct, which they can choose to inherit or change.’ To make this conceptualization usable for the historical analysis of an actor’s educational activities, this article asks to what extent the aims of citizenship appear in which context, and how they change over time.

After describing my methodological approach (Chapter 2), I will trace the evolution of the understanding of citizenship of the ITI, my case study, through three historical periods characterized by very different political and geographical contexts (Chapter 3). Chapter 4 draws conclusions and offers further considerations.

## Methodological approach to understanding rationales of education for economic citizenship

The article takes a historical approach to understand the changing and debated rationales of education for economic citizenship. Research on the history of economics education claims that school-based forms of a moral economic education were established long before a subject dedicated specifically to economics emerged in compulsory schooling (Coninck, 2012; Maß, 2018; Osborne, 2014). Economic education has often been influenced by actors outside the formal school system. Therefore, this article will not investigate economic citizenship with a particular focus on the official curriculum (e.g. by means of teaching materials or curriculum analysis). Instead, it is guided by the assumption that our dealings with economic issues shape our social relations (Garon, 2012; Maß, 2020; Meyer, 2015; Ott, 2011) and, thus, we find specific rationales of economic citizenship in the education for everyday economic interactions. This paper explores its research question with a historical case study of a private international actor. This is a case of a private interest group that has always stepped up its activities in economic education in times of crisis throughout the century.

Therefore, the article will use the perspective of an international banker association—and, in some cases, their national sub-associations—whose activities can be observed at different political levels, from the local to the international, an approach that has proven to be very fruitful in recent years, particularly for the history of education (Hofstetter, 2022; Sorensen et al., 2021). Since the international association under study has a very heterogeneous thematic focus depending on the period and geographical scope, the analysis will be focused closely on the thematic priorities of the association under study. This allows for a corresponding structuring of historical analysis and a corresponding selection of activities within this international association, in line with current debates on the role of economic education and citizenship education in times of crisis and change.

Therefore, this article traces the history of the ITI, a global banking association that has defined itself as an educational actor ever since its foundation and that has been a hub for the circulation of financial knowledge.^1^ I take observations across distinct historical periods of change and crisis—the founding of the institute in a fascist context, its reconstruction in the post-war period and its first post-colonial activities—to describe the development of different rationales of economic citizenship from the perspective of this actor.

For this reason, the article is based on extensive archival research at the ITI and its legal successor, the World Savings and Retail Banking Institute (WSBI) in Brussels. The archive of this private association is well maintained despite several moves and institutional upheavals.^2^ In addition to the documentation of the in-house journals (especially *World Thrift*) and various international congresses, the minutes and files of the specific commission on savings education and youth policy activities are documented. To close gaps, to examine local implementation of the strategies of this international actor and to cross-check the information from the perspective of the ITI sources, the archives of various (former) national members, primarily the archives of the German DSGV in Bonn were consulted.

With a research perspective that oscillates between different times and places, I will show how savings banks reached ‘beyond their counter’ (ITI, 2.6.3. USA/108, Reeves, 1927) by understanding themselves as inherently educational organizations. Arguing with Sobe and Kowalczyk (2013), the contexts under study here are not given as facts but emerge at three moments as three local focal points of the ITI’s international focus. ‘Seeing’ through this organization’s lens (Sorensen et al., 2021) sharpens our view of the rationales of its strategies and economic citizenship norms. The three periods under analysis in the development of these actors can thus be understood as a kind of microscopic case study within the ‘case’ of the ITI. It creates, inspired by Revel’s (1996) concept of *jeux d’échelles*, depth of focus, contextual relevance and specificity to arrive at more general conclusions for the observed banker association in the sense of a comprehensive case study. A case study that—also in the perception of the actors examined—revolve around three phases of crisis and societal change. In some cases, not only is the ITI as an umbrella organisation examined in detail, but the activities of national associations relevant to the case studies are also included. This again requires a selection based on the significance of these associations for the development of the ITI umbrella organisation within the periods under study.

We will see that for its founding in the 1920s, the context of early fascist Italy was of particular importance. For the new formation after the Second World War, developments in Germany were essential and, since the 1960s, the global opening of the ITI has been accompanied by a very local development aid policy in Zambia. The periods and places under analysis are therefore as close as possible to the organisation under analysis itself. From its foundation in 1924 until its peak activities in the late 1960s, the ITI showed three thematic focuses in its educational activities in three periods: direct measures in schools, curriculum development in cooperation with scientific actors and infrastructure development in post-colonial contexts (Ruoss, 2020). The article will trace the entanglement of education, citizenship, and banking in these three periods.

## Savings banks as educational actors: contingent rationales in differing contexts

Before (savings) banks could become active ‘beyond the counter’, the counters had to be installed in the first place. The establishment of savings banks from the early 19th century onward, mainly through philanthropy, was essentially an act with an educational aim. Protagonists argued that a stabilization of existing economic orders could be achieved through the financial inclusion of the working class. It was hoped that the working class’s inclusion in the banking system would combat calls for public social security and the strengthening of labour movements (Maß, 2018). The rationale of this kind of education was socialization with the aim of strengthening social cohesion. The citizen that should be educated in this context was conscientious and obedient. Schools, with the support of savings banks, became a major factor in the first period of the ‘bankization’ of industrial societies. Parents were to come into initial contact with bank counters through their children and their schools (Ruoss, 2018); children were employed here as carriers of knowledge into broader society.

### Foundation of the ITI: education, economics and citizenship in times of clashes

The ITI was founded in response to a crisis—a crisis of trust in the economic order following the hyperinflation in the early 1920s. Savings banks from 28 countries (mainly from Europe and the Americas but also from Japan and Australia) were represented at the founding congress as the first International Thrift Congress in Milan.^3^ The central aim, however, was to educate the young to become good savers, stakeholders of society and financially independent citizens, which was reaffirmed at the following congresses (ITI, Nr. 73, 1929). The ideal of the economic citizen as conscientious, thrifty and at the same time financially independent—and thus also independent of state or social support—was institutionalized in the form of the ITI in the sense of a lowest common denominator.

On the notion of citizenship, the founding context of the ITI in the interwar period is of interest, and it depended on the contexts and needs of individual members. If we look at the situation of the host country, Italy, this becomes evident. The importance of this congress takes on two forms for the Italian association. On the one hand, the driving force behind this promotion of the centenary of a local bank was an Italian attempt to normalize its fascist policy^4^ by stabilizing its foreign relations. On the other hand, the main reason for European savings bankers to join the congress was the general crisis in savings caused by hyperinflation in the post-war period.

A report by economist and Vice-President of the Savings Bank in Lugo, Achille Ballardini, for the Italian Savings Banks on the first International Thrift Congress, call for a joint educational programme are insightful (ITI, Nr. 73, 1929: 705–725). Ballardini presented a three-phased model for economic education. The first phase included teaching general and moral principles of thrift and providence (‘moral propaganda’). The second phase was called ‘economic education’ and was focused on technical economic instruments (investment products, interest, etc.). However, Ballardini claimed that improved knowledge about financial instruments alone could not anticipate or solve crises. He also felt that this technical knowledge could be used for ‘blind speculation’, which he defined as a threat to society (ITI, Nr. 73, 1929: 713). Therefore, he proposed a third phase to solve this dilemma, which was to teach ‘capitalistic education . . . as the instruction and preparation of the saver, who gradually transforms himself into a capitalist’ (ITI, Nr. 73, 1929: 713). To convey these three steps of economic knowledge to society, savings banks should be ‘transforming themselves into efficient schools of capitalistic economy’ (ITI, Nr. 73, 1929: 715). Ballardini concluded that ‘capitalistic education’ went beyond stirring emotions (phase 1) and technical knowledge (phase 2). It was about making ‘capitalistic experience, . . ., reacting against the equivocal propaganda of the many agents of dishonesty and speculation, cooperating, in short, to limit the number of the many victims of ignorance and inconsideration and to direct the people towards higher ethics’ (ITI, Nr. 73, 1929: 724f.). It was not to be corrupted by capitalism through the help of (only local or national) investments in a savings bank. In short, it was not to educate the youth for a liberal market society but to raise small capitalists in the direction of economic behaviour rooted in a nationalistic spirit (though exclusive and illiberal).^5^ This programme was explicitly meant to support indebted families in dealing with the consequences of the crisis, but it was also intended to initiate a ‘civilizational mission’ and, in his understanding, ‘ethical’ change in the political economy.

This concept of ‘capitalistic education’ mirrored a critique of capitalism common in the savings movement of the time: ‘Capitalistic education’ was not necessarily meant to educate people to live in a liberal market society. It becomes evident that some important commonalities in economic education emerged that transcended existing system boundaries, as different national associations agreed to maintain international collaboration despite system conflicts. The form of ‘ethics’ propagated here is closely related to rationales of citizenship which are social-cohesive in a nationalistic way and transformational so as to reach a future, societal goal by means of economic education. Referring Stiller’s (2018) concept of a ‘*völkisch* capitalism’, this rationale of citizenship education may be understood as transformational in the direction of a *völkisch* [national-ethnic] cohesion.

Only a few years passed between the founding of the ITI and the next major economic crisis. The third International Thrift Congress, held in Paris in 1935, was significantly impacted by the Great Depression. The primary resolution adopted by the Congress emphasised that, especially ‘in troubled times such as these we are going through at present, the principles of thrift and saving must be deeply rooted in the minds of the new generations as a fundamental factor of all normal life.’ (ITI, Nr. 74, 1935: 180). The ‘importance of systematic saving’ and the role of convinced teachers in this regard was distinctly framed as a ‘national virtue’ (ITI, Nr. 74, 1935: 1216). From the ITI’s perspective, the challenges for educating citizens therefore came less from nationalist forces than from economic theory. An internal ITI memorandum in 1938 took explicit aim at Keynes’s ‘General Theory’, describing it as ranks among the anti-thrift theories’ (ITI, Nr. 101, 1938: 1). They therefore criticize expansionary monetary policy. A countercyclical economic policy, in their view, undermine the moral and economic case for disciplined saving. Instead, the savings banks insist that capital accumulation depends on prior savings and robust monetary discipline.

The international institutionalization of the savings bank system within IT was based on a common consensus and sought to suppress possible conflict despite organizational and systemic heterogeneity of its members. The economic citizen was to support and sustain the political-economic system as an aim independent of the system itself. However, the aims of the implicit ideas of citizenship education conflicted depending on context and time. On the one hand, they clearly exhibited aspects of social cohesion. The focus on learning about economics in conjunction with the acquisition of ‘moral principles of thrift and providence’ should certainly have had a socially stabilizing effect in times of economic crises. The normalization of Italian fascism via ethically and educationally shaped economic discourses is the most striking example of the consensual strength of the economic citizen. On the other hand, the programme clearly demonstrated a transformational aim with regard to the ideals of citizenship education. The idea of ‘capitalistic education’ as a combination of experience in active financial participation in the local economy and a ‘civilizational mission’ points to the aim of a (nationalist and ethnic) transformation of society through the economic behaviour of citizens, which was to be promoted through economic education. This goal gained additional momentum with the Great Depression and the emergence of new economic theories. The education of the economic citizen thus took on new relevance. The aim was no longer simply to educate individually responsible savers, but citizens who would draw conclusions about the state from their own behaviour—and urge it to exercise monetary discipline.

### Re-thinking the citizen for the economic boom

Immediately after the war the ITI framed thrift as a ‘pre‑condition for lasting peace’, asserting that it must ‘put saving in the place it is entitled to, side by side with labour’ (ITI, Library, 1946: 4). This slogan reveals—once again—the organisation’s ongoing belief that disciplined saving rather than expansive consumption would secure both economic reconstruction and social stability. Thrift has been reactivated in its status as a moral principle. The ITI positioned economic self-restraint as a civic duty, capable of transcending political divides and rebuilding trust in institutions.

In 1947/48, the ITI was reassembled, but only by a small number of members. The most committed to re-establishment of the ITI was the German Savings and Retail Banks Association (DSGV). The spotlight on the DSGV is interesting in that, on the one hand, this national association was particularly relevant to the resumption of ITI as umbrella organisation’s youth policy mission and, on the other hand, it had to deal with a fundamental crisis of legitimacy after the war and its involvement in National Socialism (Ruoss, 2020). The DSGV was engaged in dealing with the overall political crisis and the creation of new citizenship norms. This can be seen, for example, in the fact, that at the DSGV’s World Savings Day ceremony in 1952, the first German federal president, Theodor Heuss (1952: 11), gave a speech arguing that ‘people who save want to be free’. Capital formation was not a final goal but instead sought to provide ‘education to economical, thrifty people’ (ITI, 1952: VII). The DSGV’s conception of citizenship nevertheless remained culturally conservative. Dual-earner couples and early marriages were taxed by congress organizer Rudolf Heindel as the epitome of materialism (ITI, Nr. 537, 1964, 3/4). Not least, the DSGV provided a platform for savings education ideas from former National Socialists such as the youth book author Lydia Knop-Kath (1963a, 1963b) or the former professor of special needs education and rector at the Pedagogical Institute in Karlsruhe Josef Spieler (1957). Heindel (1932) himself had also previously published works on National Socialist economic theory.

The tone of the DSGV’s publications remained focused on thrifty economic citizens as the backbone of the new political order. Whilst the 1959 conference on savings education in Frankfurt a. M. concentrated on the evolution of a market economy that regarded education as the cornerstone of economic advancement, it also invoked a neo-classical topos with the economic principle of utility maximisation. It is evident that Keynesian counter-narratives are absent from the debate. Instead, an (ordo-)liberal form of moralising consumption and debt dominates (ITI, Library, 1959). What we see here is a modification of the rationales of citizenship education, aimed exclusively at social cohesion and a form of socialisation of a conscientious and obedient citizen. Basically, we see a continuity of educational activities with more conservative goals for economic education. The nationally cohesive and transformational rationales of citizenship education were overwritten by earlier forms of social cohesion.

From this perspective of the German association, we can in turn take a look at the discussions at the ITI level. As keynote speaker at the ITI’s International Savings Banks Congress in Vienna in 1963, economist Friedrich von Hayek was invited to talk about the importance of economic education and individual experience with capital to stabilize capitalist economies (ITI, Nr. 76, 1963). The enthusiastic welcome of a neoliberal apologist at one of the most important global gatherings of savings and economic educators is emblematic of educational attempts to mould ‘consumer citizens’ since the 1960s. The development of the consumer citizen figure was closely related to the assertion of economic theories, which, in turn, understood knowledge as capital. Even Rudolf Heindel published an essay entitled ‘Investment clubs for young people’ in 1970 (DSGV, Sparkassen Werbedienst, 1970/7). Intellectually, this interpretation of the period of ‘emancipation’ of the citizen from the political in the economy and the reduction of economic actors to ‘good’ consumers was accompanied by other references (Anger, 1982). As a central mode of learning by economics, the experience of dealing with capital is placed at the centre. Experiences in youth investment clubs are in turn oriented towards underlying attitudes regarding the rationales of citizenship. Social integration should thus be possible primarily through economic success rather than through naturalization, which needs to be contextualized within the rising mass consumer societies. The structurally conservative element remained in the new guise of economic education as consumer education, even though the self-portrayal referred to an emancipatory phase. The danger of mass consumerism needed economic education as a counterbalance that should be achieved not only through moral appeals but also by learning how to manage money and consume sensibly.

In summary, in the first post-war decade, a consensus again prevailed that savings education should primarily contribute to system stabilization and social cohesion. However, the aims of citizenship education were not always consensual but involved conflict. There was a conflict of values, particularly concerning the family and the economic status of its members. A consensus emerged in the 1970s under the pressure of stronger marketization of the savings banks. Here, the formal argumentation of traditional sufficiency also led to the promotion of the economic citizen as individually responsible for its consume, and at the same time, this argument was also used to justify general deregulation and a stronger customer focus on the part of the savings banks themselves.

### Old recipes for a post-colonial context

In spring 1968, following a pledge from the United Nations (UN) Economic Commission for Africa (ECA), Milan professor of economy and consultant with ITI Arnaldo Mauri, and the deputy director of the ITI in Amsterdam, Johann G. Wallis de Vries, went on what they called a ‘scouting expedition’ to a number of east African countries (ITI, Nr. 136, 37, Sep. 1968). Their declared aim was to look out for ‘appropriate’ countries in Africa to promote their ideas about the economy and economic education (ITI, Nr. 136, 35, 1967). This aim presupposed that it was possible to generate declarations of intent from actors in African countries, support from other international organizations and commitment from their own institutes. This happened from the late 1960s, when non-European members started to demand more participation within the ITI. For example, a first seat on the ITI board of administration for a delegate of an African member was created in 1969. What happened in this context was the beginning of the ITI’s active involvement in the development aid policies of large international organizations. The ITI and the national associations became players in the post-colonial power struggle. The article’s focus for this third period is therefore on the subject area that strongly drove the ITI’s educational activities in these years.

The ITI delivered technical training to banking staff but went beyond this by making educational efforts to change population behaviours and perceptions of the economy. Mauri’s memorandum (ITI 2.5.3. I/ACRI/0014, Mauri, 1969) was clear in this respect: as it was ‘not always easy to make a radical change in adult habits and behaviour’, thrift should be promoted among the young and the ‘main effort should be made in schools’. With both internal and external claims for a stronger focus on Africa and due to arguments for market competition, already in 1967 the International Savings Research and Operating Program was proposed. The programme proposed to the ITI National Associations Committee contained four steps: (1) an ITI delegation bargains with national ministries in African countries; (2) ITI experts develop the practical part of the plan; (3) employees in African institutions are trained in Europe or the United States; and (4) ITI experts stay and control the development for the first two years (ITI, Nr. 215, 1967).

Mauri and Wallis de Vries’ ‘scouting expedition’ was only the first of such visits and was followed up in early 1969. Their results were presented at the UN ECA Meeting on Technical and Social Problems of Urbanization in Addis Ababa. Two consequences can be highlighted. First, the ITI entered the world of the UN, and the United Nations Development Programme (UNDP) started to finance the ITI with its technical assistance service in Africa. Second, the ITI closed its ranks towards development cooperation policy, establishing its own committee on development aid. This happened against an older engagement in colonial banking and a hesitant policy during the first post-colonial decade. It was more than just lip service, as the ITI decided to move its headquarters from Amsterdam to Geneva, which was considered essential ‘in this day and age, if the Institute is to play a full part in international affairs’ (ITI, Nr. 136, 1969, appendix to the minutes). After the presentation of its reports in Addis Ababa, Zambia was chosen as a ‘good test case’ for savings promotion and the institutionalization of savings institutions in Africa (ITI, Nr. 136, 1968). The country had already been conducting a savings campaign since 1966, and it was the first to officially ask the ITI for support, with the head of the Zambian Postal Bank visiting the institute’s headquarters in Amsterdam (ITI, Nr. 136, 1968).

A third ‘expedition’ was organized with the financial and organizational support of the UNDP. Hans Greisinger, a clerk with the Austrian Savings Banks Association, went to Zambia for a six-month study period. As he lacked any professional international experience, he had a stopover at the ECA in Addis Ababa for a ‘three day pre-mission briefing’ (ITI, Nr. 244, 1970). Despite Greisinger’s inexperience, the collection and interpretation of data and the drafting of recommendations relied on his field survey. His starting point was the observation that declining savings rates and reasons for low savings rates were founded on several cultural patterns: ‘The main factors working against increased savings in the economically active regions are socially determined and are rooted in the impact that economization and its concomitant phenomenons [sic] registers on broad strata of the Zambian population’ (ITI, Nr. 244, 1971: 3). Greisinger blamed traditional forms of social support such as informal credit practices (‘chilimba’) as part of a culture that he perceived as hostile to individual saving. High-interest or well-established financial institutions would not attract the ‘small African saver’ (Greisinger, in Wallis de Vries et al., 1972: 55). The report concluded, that ‘from the savings habits of the Zambian adults confirms the assumption that economic education within the Zambian families tends rather to the emphasis placed on the satisfaction of wants of the moment.’ (ITI, Nr. 244, Report on Savings in Zambia, n.d.). While Greisinger did not delve deeper into an understanding of this ‘economic culture’, his observations were made from a perspective of what is measurable and commodifiable. Again, the main solution was framed as educational: ‘The present savings attitude is mainly a consequence of lost educational occasions in the past. The attitude to plan is teachable, and the Zambians are well-known for their zeal for learning’ (ITI, Nr. 244, 1971: 38, original emphasis). A conflict was emerging regarding the aims of education for economic citizenship. On the one hand, there were existing forms of social cohesion through economic practices. On the other hand, the ITI actors in this case did not represent a position of social cohesion, but of social transformation. The ITI’s Eurocentric ideal of social cohesion was always associated with institutional forms of financial operations, which differed from the less individualistic and more informal and traditional economic practices in these ‘test cases’.

This assessment led to recommendations pointing to compensation for the so-called ‘lost educational occasions in the past’, which only hypothetically referred to Zambian history, but more rhetorically to the historical roots of the ITI. According to the report, the main objectives should be twofold. First, a national savings bank (the National Savings and Credit Bank, NSCB) and, thus, the structures for long-term savings should be established. Second, this savings bank should be made active in myriad ways, including in savings education at all school levels, with school savings schemes, with participation in World Thrift Day, lectures and seminars for teacher training, teaching materials (primers, economic bulletins, school journals, posters, films), working groups with teachers, sightseeing visits to NSCB branches, conferences for young people and with local youth associations, social events for young people, and with youth magazines (ITI, Nr. 244). Educational issues were a priority. Savings institutions and schools should establish an economic culture and a savings habit to make savings institutions, and the entire national economy work differently. Yet, despite the detailed nature of the survey of the situation on the ground, the proposals were no more than a summary of the experience of the past 50 years regarding savings education among ITI members.

In May 1971, the Zambian government agreed with Greisinger’s recommendations. Delegated by the Zambian Ministry of Finance and organized by the ITI, the head of the Zambian Postal Savings Bank, Jacob Nyoni, travelled through Europe for 3 months to receive training at different banks (ITI, Nr. 10, 1971; ITI, Nr. 244, 1971). Although the NSCB was established and some promotional and educational activities were launched, the long-term impact of their effort remained – in their view – unsatisfactory. Not even membership of the NSCB in the ITI succeeded before 1985; both the Zambian government and the NSCB aborted the contact unilaterally (ITI, Nr. 245, 1974 and 1975).

Education for development was not limited to financing schools and general education. It also included deploying highly specific content and knowledge about the economy and society. To ‘develop’ in this context meant to overcome traditional forms of social and economic behaviour and to distinguish a field of (monetarized) economics from other social practices (for Africa, see e. g. Cooper, 2019; Unger et al., 2014). It was to spread the liberal belief in the individual’s responsibility and potential for promoting the common good by primarily following one’s own interests. The means to deploy such a ‘change of cultural patterns’ were seen as the very same that had been used since the 19th century to integrate the working class into a liberal market society. It was the confrontation of theoretical conceptions with familiar practices which emphasized education as a central means by which to enforce a market-driven economic system – and to keep blaming educational and cultural patterns for economic failure. Personal savings were recognised as the pivotal catalyst for both private and public investment and growth. This assertion is predicated on a fundamental postulate of neoclassical economic theory. The state should be content to create the framework conditions (e.g. savings campaigns, technical assistance), but should refrain from stimulating demand through expansionary fiscal policy in the Keynesian sense, for example. This underlying attitude permeates the entire non-European ITI policy. This is also evident from analysing vast amounts of archived minutes and reports from the ITI Development Cooperation Committee from the following decade. The primary function of the state is to establish institutions that promote savings, such as in educational institutions and through savings banks.

As Ydesen and Grek (2020) showed with regard to the Organisation for Economic Co-operation and Development (OECD), when education became a policy in their ‘struggle for organizational survival’, the savings banks did not have to invent an educational policy. Rather, they had to transfer their historically well-established practices into new contexts. The forms of knowledge that emerged from these practices remained completely tainted by the historical experiences of the actors involved. From school savings schemes to World Thrift Day, these were concepts that echo claims from another time, when savings banks were seen as individual, educational solutions in a context of industrial societies, and at the forefront against the development of a welfare state. Once again, system building and stabilization were understood as objectives of institutionalized savings, in this case with a time lag and from the perspective of mainly European actors. A continuity of rationales of citizenship can therefore be observed. However, depending on the context, this continuity conflicted with the aims of citizenship education. The aims of the ITI in its activities in Zambia were primarily transformational in that they were intended to create a form of economic citizens as customers and youth as a market.

## Conclusions and outlook: economic education for banks or citizens?

This article has explored the rationales of citizenship in the development of economic education using the case of an international private interest actor in the banking sector. By doing so, it shows that the history of savings banks and their international activities can be read as part of the history of citizenship and citizenship education. The article traces how the ITI reacted to some of the major crises and changes it encountered during its existence. Despite the different contexts in which these crises and societal change took place, this bankers’ association advocated relatively stable forms of economic education, and particularly a very stable set of rationales of citizenship education. At the same time, while there is a high degree of continuity in the socially cohesive rationales of citizenship education championed by the ITI, there are also time- and context-specific differences.

The first period—the foundation of the ITI in a context of conflict and financial crisis—highlights how the international institutionalization of the savings bank system was based on a common consensus and mission to suppress potential political conflict around the role of savings banks in (il-)liberal societies. The prevailing agreement remained on the paramount importance of education and the imperative to ‘conquer the minds of the young’, despite different countries’ organizational and systemic diversity. In this context, the economic citizen was expected to uphold and perpetuate a country’s political-economic system as a goal separate from the character of the system itself. The example of the Italian Savings Banks Association is a case in point. They advanced a nationalist critique of the liberal economy and society through a comprehensive concept of economic education that combined knowledge, skills and attitudes. From this perspective, economic education—‘capitalist education’ in the literal sense—was intended to achieve anti-liberal goals, namely a form of *völkisch* cohesiveness that would transform society in a nationalistic way. Although this rationale of citizenship was not universally shared by members of the ITI, the organization did provide it with a prominent platform. This goal of social cohesion by individual savings gained additional momentum during the Great Depression, when new economic theories emerged. The education of the thrifty citizen became even more relevant in this context. The aim now was to educate citizens who would draw conclusions about the state based on their own behaviour, urging it to exercise monetary discipline – not just to educate individually responsible savers.

The second period under study—the decades following the Second World War—shows a renewed consensus within the ITI that economic education should primarily contribute to the stabilization of the system. Economic theories of reference had not changed insofar as deficit spending is concerned. Economic citizens should continue to view debt as a moral issue, in their capacity as consumers, savers and political citizens. Hayek’s appearance at the 1963 International Savings Banks Congress was no coincidence; it was consistent with the idea of encouraging economic citizens through education. However, different rationales can be identified within the association under study. Despite the persistent emphasis on the economic education of social cohesion, the association has progressively adopted rationales with individual-emancipatory aspects into its positions. The latter became particularly relevant in the 1970s with the transformation of savings banks themselves, driven by their stronger market orientation. In this context, former arguments for traditional thrift were extended to market-like efficiency as the objective of financial education, aligning with the ideal of the consumer citizen.

The third period under study once again reveals another shift in the ITI’s understanding of economic education, which was once again perceived as means of constructing and stabilizing political systems. These rationales can predominantly be described from the perspective of European actors. Within the ITI, a consensus existed on the need to strengthen the association’s role within the sphere of international organizations through its (international) educational commitment. However, if one considers the influence of this commitment on the ground, limits and conflicts become apparent. It is unclear to what extent the short existence of ITI activities in Zambia was related to rationales of citizenship education. From a European perspective alone, we can only analyse the change in ITI activities. These activities, however, reveal a clear conflict concerning the aims of citizenship as emphasized by the ITI itself. The ITI provoked a conflict between its rationale of transforming society by educating youth to adopt individualistic economic behaviours as consumers and existing local informal economic practices oriented towards social cohesion. In this context, ‘developing’ economic citizenship meant overcoming traditional forms of social and economic behaviour and distinguishing a field of monetised economic interaction from other social practices.

Which rationales of economic citizenship were put forward in times of change and crisis? Following the history of the ITI, the production of economic citizens appears as a history of a juxtaposition of underlying conflicts wrapped in a constant need for consensus to stabilize political systems. Rationales range from a broad understanding of ‘capitalistic education’ as an experience-based strengthening of emotional reflexes in dealing with money, with the aim of promoting social cohesion, to an understanding of economic education as a transformational intervention aimed at generating citizens as customers and to convert traditional forms of economic exchange into institutionalized saving. The consistent rationale throughout the various contexts and crises is the personal responsibility for one’s own financial situation and for achieving financial balance in society as a whole. From this perspective, the primary function of the state is to establish institutions that promote savings, including schools and their links with savings banks. This rationale had a long tradition within the ITI’s efforts to promote economic education. It was not reinvented with the emergence of neoliberalism. Rather, neoliberalism allowed the organization to resurrect the rationale of thrift education from its founding period. Neoliberalism thus re-legitimized the understanding of citizenship education held by organizations such as the ITI that never viewed democracy and democratic participation as goals of education, but rather individual economic participation. What has been called an ‘economic shift in thinking about youth citizenship’ (Meylemans et al., 2023: 16), starting with the banking crisis in 2008, reaches further back in time if we take a perspective that considers private interest groups acting beyond the nation-state.

This does not mean that the history of this actor should be understood as a history of economic education as such. Heterodox forms of education for economic citizenship, such as those that conflict with ITI policy in non-European contexts, or cooperative ideas of a democratic economic citizenship, for example, are not reflected in saving banks’ perspectives. Therefore, this analysis may say more about the change in the educational understanding of a specific private interest group from the financial sector than it does about the locally very different and not universal development of different concepts of education for economic citizenship. The actor and the source-driven selection of the three periods under study clearly show the changing approaches of this international organization to educational issues. This strategy involves limitations when it comes to interpreting statements on the (local) effectiveness of the organization’s international efforts. From the ITI’s perspective, none of its efforts seems to have led to a coherent and successful local implementation of its international policies. But to understand local heterogeneity in the implementation of the ITI’s rationales, a different, more micro-analytical perspective would be needed.

What further avenues of research does this analysis suggest? Economic education obviously does not only serve to educate (different) ideals of economic citizenship. Economic education was used throughout the 20th century as a means of legitimizing economic actors themselves and stabilize their position. It remains a standard response of private interest groups when mismanagement and financial crises hit large parts of society. The role attributed to education by these and other actors as the foundation of economic citizenship is further evidence of the great consensual integrative power of education as a solution. The bank counters, in the sense of experiences with money and investments, and the desks of teachers, as simulation and as multipliers of financial knowledge and financial skills, are addressed side-by-side and understood as mutually necessary. When, theoretically, consumption is not simply the economic counterpart of production, but is understood as a central means for the productivity of the entrepreneurial self (Foucault, 1979), the consumer citizen is primarily constituted by their everyday financial activities.

Against the background of the historical development of such activities, research and practise in citizenship and economic education should consider what emphasis is placed on its contribution to the further development of the economy and society for social cohesion, individual-emancipatory and transformational aims. If we adopt a standpoint that acknowledges the significance of these normative aims for citizenship and economic education in developing a democratic and egalitarian society, then we must recognize that this type of education should not serve the agenda of private interest groups. Economic citizenship must therefore remain a broad and controversial concept to raise questions about the economy, and thus about systemic issues. These are issues that cannot be addressed through individual behaviour, but rather through political action. Economic citizens need to recognise political agency, collective deliberation, and structural conditions.

## Notes

1. The International Thrift Institute (ITI) was renamed the International Savings Banks Institute (ISBI) in 1963. In this paper, the abbreviation ITI is used for the entire period up to the restructuring to the World Savings and Retail Banking Institute (WSBI) in 1994.

2. My thanks go to Nancy Lockkamper, who has repeatedly shown great willingness to provide me with a wealth of material from the archives.

3. Although there are still very few historical studies on this actor, this article is based on a report (Ruoss, 2020) that describes the activities and sources of the ITI in greater detail from the perspective of circulation of knowledge. This allows me to examine the three phases of the crisis and change from a specific theoretical perspective of economic citizenship. To make this analysis comprehensible, I would like to provide a few descriptive contextual details that were not included in the above-mentioned report: In the decade following its foundation, membership figures initially grew only slowly but increased again more strongly after the Great Depression. By the start of the Second World War, for example, associations from British India, Dutch East India, New Zealand, South Africa, Argentina and Brazil had also joined. Only nine European members were initially involved in the new foundation in 1948. By 1949, according to annual reports, the number had grown to 46 (Europe 33, South America 6, USA 1, Asia 6). In 1970, the ITI had 67 members (Europe 44, South America 5, USA and Canada 2, Asia 6, Africa 5, Oceania 5) and by 1992, the year of the largest restructuring, 125 members. The first African members were five former colonial savings banks in 1970 (Algeria, Burundi, Kongo, Swaziland, Rwanda). The 1970s were a decade with a big growth of African members (up to 21 in 1981). In the 1980s, African membership continued to grow, but growth was also seen rapidly among South American members, up to 22 in 1992. After the early 1990ies European, American and Oceanian membership declined, African membership persisted, and Asian membership grew. We could therefore speak of two European decades (1940s–1960s), an African decade (1970, with permanent growth until member majority), an American decade (1980s) and an Asian decade (1990s).

4. Benito Mussolini was Honorary President of the Congress. At the inaugural ceremony he was ‘greeted by an outburst of applause’ by the 2,000 guests attending (ITI, Nr. 72, 1924: 135).

5. Especially in the case of Nazi Germany, savings banks presented themselves as financial institutions close to the people in order to distance themselves from high finance, which was charged with anti-Semitic attributions at the same time (Muschalla, 2018; Salden, 2019).

## Archives

ITI Archives, Brussels:

ITI, Nr. 10, Report of Activities, 1971–1981.

ITI, Nr. 72, First International Thrift Congress, Report, Milan, 1924.

ITI, Nr. 73, Second International Thrift Congress, Report, London, 1929.

ITI, Nr. 74, Third International Thrift Congress, Report, Paris, 1935.

ITI, Nr. 76, Seventh International Savings Banks Congress, Vienna, 1963.

ITI, Nr. 101, Meeting of the Permanent Committee, Stockholm, 1938.

ITI, Nr. 136, Meetings of the Board of Administration, Minutes, Sep. 1968 & Jan. 1969.

ITI, Nr. 215, National Associations Committee, 1966–1970.

ITI, Nr. 244, Development Cooperation Committee, 1970–1971.

ITI, Nr. 245, Development Cooperation Committee, 1972–1977.

ITI, Nr. 247, Development Cooperation Committee, 1980–1981.

ITI, Nr. 537, Contact. International Review on Savings Banks Publicity, 1962–1968.

ITI 2.5.3. I/ACRI/0014, Mauri A (1969) The Promotion of Thrift and Savings in Developing Countries.

ITI 2.6.3. USA/108, Reeves MB (1927) Beyond the Counter.

ITI Library. Pädagogische Arbeitstagung in Frankfurt am Main. Am 26. und 27. Juni 1959.

ITI, Library, The International Thrift Institute for the Savings Banks, for Thrift, for Peace, 1946.

DSGV Archives, Bonn:

Internationales Institut für Sparkassen [ITI] (1952). Tagung für Sachbearbeiter des Jugendsparens. 5. und 6. November 1952. Stuttgart: DSGV.

Deutscher Sparkassen- und Giroverband (DSGV) (1966). Sparkassen Werbedienst.
